# Assessment of *Pleurotus ostreatus* and *Pleurotus citrinopileatus* mushroom as bioshielding materials for neutron attenuation in medical cyclotron

**DOI:** 10.1371/journal.pone.0362063

**Published:** 2026-11-04

**Authors:** Md. Jahid Hasan, Md. Nahid Hossain, Hasan Mehdi, Jannatul Ferdous, Md. Shohag Mia, Md. Akhtaruzzaman, Md. Mohi Uddin

**Affiliations:** 1 Department of Nuclear Engineering, Chittagong University of Engineering & Technology, Pahartali, Chittagong, Bangladesh; 2 Cyclotron Division, National Institute of Nuclear Medicine & Allied Sciences (NINMAS), Bangladesh Medical University Campus, Shahbag, Dhaka, Bangladesh; 3 Health Physics Division, Atomic Energy Centre, Bangladesh Atomic Energy Commission, Dhaka, Bangladesh; 4 Department of Chemical Engineering, Faculty of Engineering, Islamic University of Madinah, Madinah, Saudi Arabia; 5 Sustainable Research Center, Islamic University of Madinah, Madinah, Saudia Arabia; 6 Department of Physics, Chittagong University of Engineering & Technology, Pahartali, Chittagong, Bangladesh; 7 Advanced Materials Research Laboratory for Energy Storage Capacity Enhancement, HEAT Project (SPP-13266), Department of Physics, Chittagong University of Engineering & Technology, Pahartali, Chittagong, Bangladesh; SVM Arts, Science and Commerce College, INDIA

## Abstract

Neutron absorbance efficiency is crucial for radiation shielding and nuclear reactors, with mushrooms like Pleurotus Ostreatus (PO) and Pleurotus Citrinopleatus (PC) enhancing this property due to their nitrogen, phosphorus, potassium, cadmium, and carbon content. Processed into 3 × 3 inch blocks at the National Institute of Nuclear Medicine and Allied Science (NINMAS), the mushrooms are dried at 80°C and ground to powder for analysis. Structural studies using XRD, FESEM, FTIR, and UV-vis spectroscopic reveal high carbon and oxygen levels. For the PO, the XRD investigation showed a broad diffraction peak that corresponded to an estimated crystallite size of about 1.34 nm, while PC showed a mostly amorphous structure devoid of clear crystalline diffraction peaks. The FTIR identifies various functional groups, and UV-vis spectra confirm the presence of sulfur and carbon. Neutron interaction mechanisms in mushroom-derived shielding materials involve moderation and scattering by the hydrogen-rich matrix, followed by capture at nitrogen and sulfur sites. HPGe spectroscopic confirms effective neutron interaction and attenuation via the detection of activation products such as K-40 in this study. Both samples show significant K-40 levels of 30 Bq/kg, while Th-232 and U-238 are found to be 2–3 Bq/kg and 5–6 Bq/kg, respectively. The findings demonstrate that these mushroom blocks are not neutron-transparent, but rather contribute to neutron moderation and absorption acts as a bio-shielding materials in the cyclotron facility.

## 1. Introduction

The increasing usage of nuclear energy over the past few decades has been very beneficial to the planet. Nuclear energy is a sustainable and ecologically friendly energy source that is essential to the production of power worldwide. It generates around 10% of global energy production and emits fewer greenhouse gases than many other energy sources [1,2]. Neutron absorption efficiency is the efficiency of a material in absorbing neutrons, influenced by neutron energy and material composition. It is often measured by the neutron absorption cross-section, with applications in neutron detection and nuclear reactor control. The higher the neutron absorption capacity of materials, the stronger those materials can act as shielding materials. Since neutron absorption efficiency depends on the amount of *B, Cd, Gd, Li, Sm, In, Hf, Dy* and *Eu* in a material, good shielding materials must contain these substances. The nuclear radiation shielding material is crucial for particle accelerators and reactors, but its use poses significant health risks due to potential damage to essential organs [3–6]. Concrete is a popular shield material due to its affordability and adaptability, with its radiation shielding properties influenced by its composites, including iron and boron carbide [7]. At photon energies of 59.5 and 661 keV, twelve concrete samples were tested for their shielding against gamma and neutron radiation using the total mass attenuation and linear attenuation coefficients, atomic cross-sections, effective atomic numbers, half-value thicknesses, and effective electron densities. The results showed a fair degree of agreement, but there was a shift in composition and energy [8]. The FLUKA Monte Carlo simulation [9,10] code was utilized to analyze the properties of ferro-boron and boron carbide that were used as shielding materials in concrete shields [11], concrete’s polymers provide neutron radiation protection [12] and concrete reinforced with biochar has the potential to be a neutron shielding material [13]. Neutron transport models are used to evaluate Zr(BH_4_)_4_ and ZrH_2_ as advanced shield materials; Zr(BH_4_)_4_ has a higher hydrogen density but a worse neutron shielding effectiveness. Steel and Zr(BH_4_)_4_ increase neutron shielding capacity, whereas combinations reduce shield thickness by 19% and 6.5%, respectively. In fusion reactors, boron influence reduces radioactive waste (radwaste) by lowering neutron flow [14]. The fast-neutron shielding behavior (FNSB) of ball clay and kaolin clay materials from southwest Nigeria was examined in another investigation. Metrics including mean free path, mass removal cross section, and effective removal cross section were calculated using the Win NC-Toolkit. Material availability, thermal stability, and water retention were taken into account when comparing the FNSB of the clays to ordinary concrete [15]. Evaluated the neutron shielding capabilities of polyester-matrix composites reinforced with 10–50% boron carbide, finding that thickness and B_4_C content improved neutron attenuation, but effectiveness declined above 40% B_4_C [16,17]. There is a study about X-ray and gamma radiation shielding factors in aluminum silicon alloys and neutron shielding characteristics. According to the data, ferro-silicon alloy has a maximum exposure accumulation factor, a minimal mean free path, Half-Value Layer (HVL) and Tenth-Value Layer (TVL), and is an excellent absorber of x-ray and gamma radiation. It is the most effective shielding material for neutrons due to its huge attenuation characteristics [18]. In order to attain a thermal neutron transmission ratio of less than 10^−5^ for a natural Gd content of 0.6 at. %, the particle size of the Gd-rich phase should be kept under 20 μm that confirms in a neutron shielding study for a gadolinium doped nickel alloy. Research on the thermal neutron shielding qualities of rare-earth (RE) nickel alloy materials shows that adding RE elements—especially Eu and Dy significantly improves the alloys’ ability to shield against thermal neutrons while lowering secondary gamma harm by radiation and irradiation [19,20]. In most of the cases, mushrooms are excellent nutritional sources since they provide proteins, fibers and mineral, such as K, P, Fe and neutron spectrometer [21,22]. Besides the edible properties of mushroom, it uses as radiation shield. The study explores the potential of melanin, derived from black edible mushrooms (*Auricularia auricila-judae*), as an efficient oral radioprotector. Mice fed these mushrooms before undergoing 9 Gy total body irradiation demonstrated significant protection against radiation, indicating their potential development into oral radioprotectors [23]. The study evaluates the effectiveness of 0.5 kGy X-ray irradiation for preserving the sensory attributes and physicochemical properties of shiitake mushrooms, suggesting it as a viable method to prevent spoilage [24]. The polysaccharide protein complex (PPC-Pr) from the mushroom Phellinus rimosus exhibits protective effects against oxidative stress from gamma radiation. Treatment with PPC-Pr significantly improved antioxidant levels and comet assay parameters, demonstrating its antioxidant and DNA protection capabilities [25]. The microwave absorption properties of Morchella Esculenta were studied through its dielectric and morphological behavior, showing effective absorption in the 2–18 GHz range. Notable resonance peaks began at over 5 mm thickness, with a significant peak at 13.78 GHz showing a reflection loss of −27.37 dB at 7 mm thickness and a bandwidth of 8.15 GHz. The material’s structure, including plates and wires, contributed to polarization and conductive losses, further enhanced by carbon and oxygen, indicating its potential for good microwave absorption [26]. The study on Morchella Esculenta identified microwave absorption characteristics in the 2–18 GHz frequency range, noting resonance peaks at thicknesses over 5 mm, particularly a peak at 13.78 GHz with a reflection loss of −27.37 dB at 7 mm and a bandwidth of 8.15 GHz. Structural features, including plates and wires, contributed to losses from polarization and conductivity, with carbon and oxygen enhancing its microwave absorption potential [27].

Mushrooms which are rich in bioactive compounds, including polysaccharides, and their structural and physicochemical properties have attracted interest in biomedical and radiation-related applications [28–31]. And, it is evident that most cases mushroom used as shielding and edible properties, there is no report on neutron absorber as bio-shielding in the cyclotron vault room yet. In this study, we are intended to evaluate neutron absorbance efficiency of *Pleurotus Ostreatus* and *Pleurotus Citrinopleatus* mushrooms and to be used them as shielding materials in a medical cyclotron vault room.

## 2. Experimental details

### 2.1. Sample collection

There are two varieties of powdered mushrooms *Pleurotus Ostreatus* (PO) and *Pleurotus citrinopleatus* (PC) were collected from the Bangladesh Mushroom Development Institute at Savar, Dhaka. The powders are oven dried and made finer by hand milling. The block diagram outlines of the experimental procedure for preparing and analyzing mushroom (PO, PC) samples is depicted in Fig 1.

**Fig 1:**
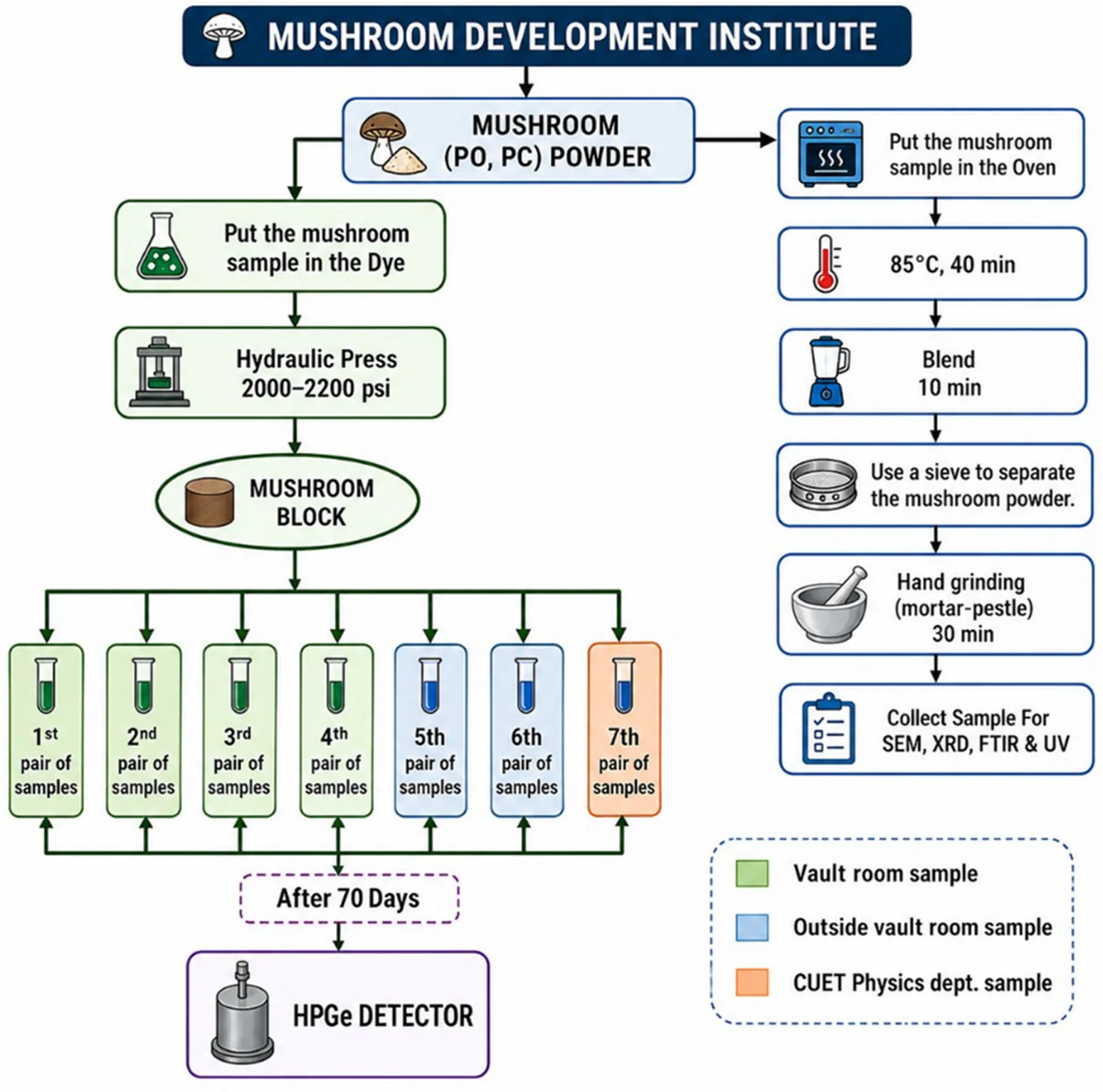
Block diagram of complete samples preparation and experimental procedures of PO and PC mushrooms.

The sample preparation process divides into two sections. In the 1^st^ section, samples are prepared for the structural, morphological and functional group analysis. In the 2^nd^ section, samples are arranged for the measurement of neutron absorbance using the HPGe detector.

**Section- 1 (Mushroom Powder Analysis):** The mushroom powders are manually mixed, sieved, and fine powdered after being oven-dried for 40 minutes at 85°C. To make the powdered mushrooms drier, we kept them in the oven for a certain time and at various temperatures. For forty minutes, the two kinds of samples were maintained at 85 degrees. They were then sieved after blending for 10 minutes. To make the mushroom powders finer, they were pounded for 30 minutes in a mortar and pestle by hand milling. The samples were then separated into small sample bottles (Fig 2). The obtained fine powder is subjected to SEM, XRD, FTIR, and UV spectroscopy for structural and chemical study. Second, we made samples for UV, FTIR, XRD, and FESEM. The samples subjected to a UV-visible spectrometer, FTIR (Fourier Transform Infrared Spectroscopy), XRD (X-ray Diffraction, Cu-Kα radiation and λ = 1.5406 Å), and FESEM (Field Emission Scanning Electron Microscopy). The structural analysis of the samples is performed by the XRD and morphological study has also been completed using FESEM. The absorbance spectra of the mushrooms at various energy have been observed by the UV-vis spectroscopy. The functional groups of studied mushrooms PO and PC are analyzed by the FTIR. All measurements are performed in the ambient temperature.

**Fig 2:**
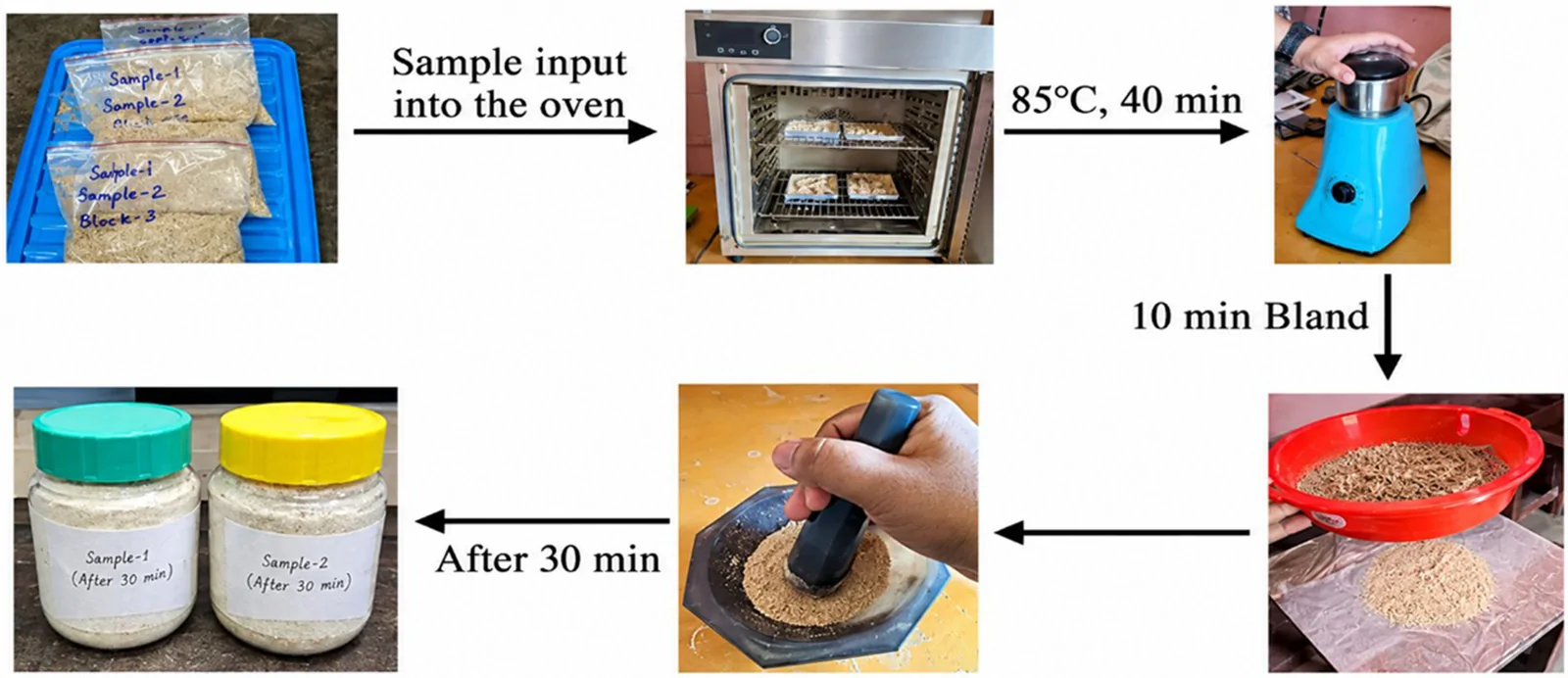
Sample preparation process for FESEM, XRD, FTIR & UV-vis spectrospcopy.

### 2.2. Characterization techniques

The structural analysis was performed by the X-ray diffraction (XRD) spectra of the synthesized samples with the Rigaku Smart Lab diffractometer (Cu-Kα radiation and λ = 1.5406 Å). Scanning electron microscopy (SEM) (JEOL, JSM6010LA) and transmission electron microscopy (TEM) (TALOS F200 G2, Thermo Fisher Scientific, USA) was employed to study surface morphology and particle size distribution. A Fourier-transform infrared spectrophotometer (FT-IR) (Jupiter, STA449 F3) was used to analyze the vibrational modes of the synthesized samples. The bandgap was also estimated using UV-vis diffuse reflectance spectra (UV-DRS) (LAMBDA 1050, PerkinElmer-USA).

**Section-2 (Mushroom Block Preparation):** To create blocks, the powdered mushroom is compressed between 2000 and 2200 psi and 3 × 3-inch blocks with the help of customized die by the hydraulic press (Fig 3). Seven pairs of samples are placed: two outside the vault, fours in the vault room, and one in the CUET Physics Department as shown in Fig 4. The samples are kept for the 70 days.

**Fig 3:**
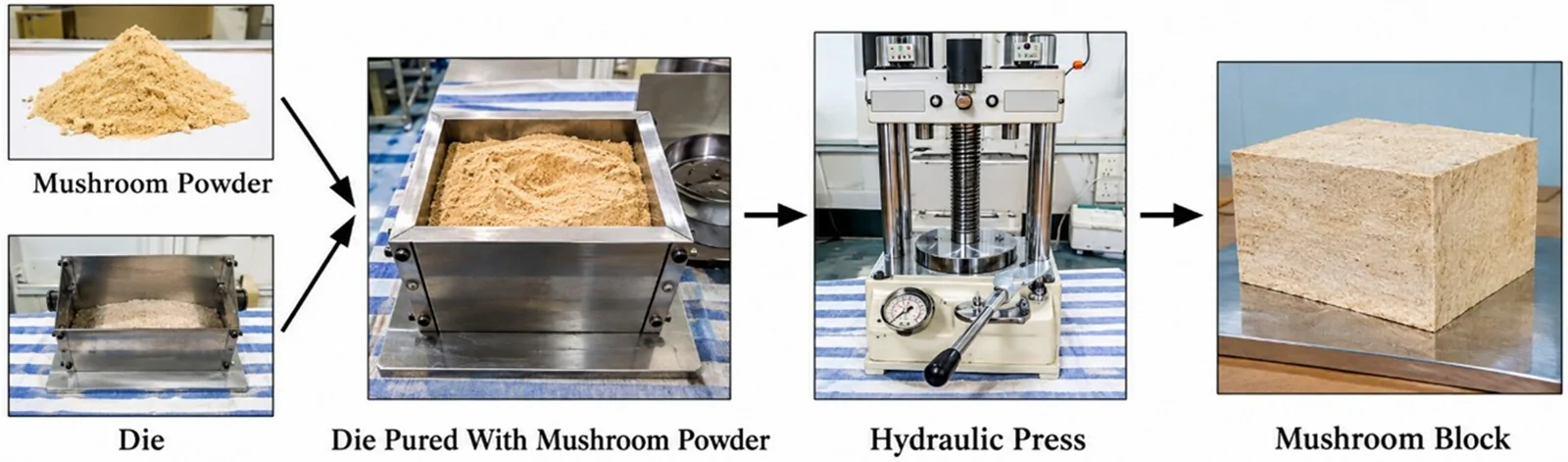
Mushroom Block making process.

**Fig 4:**
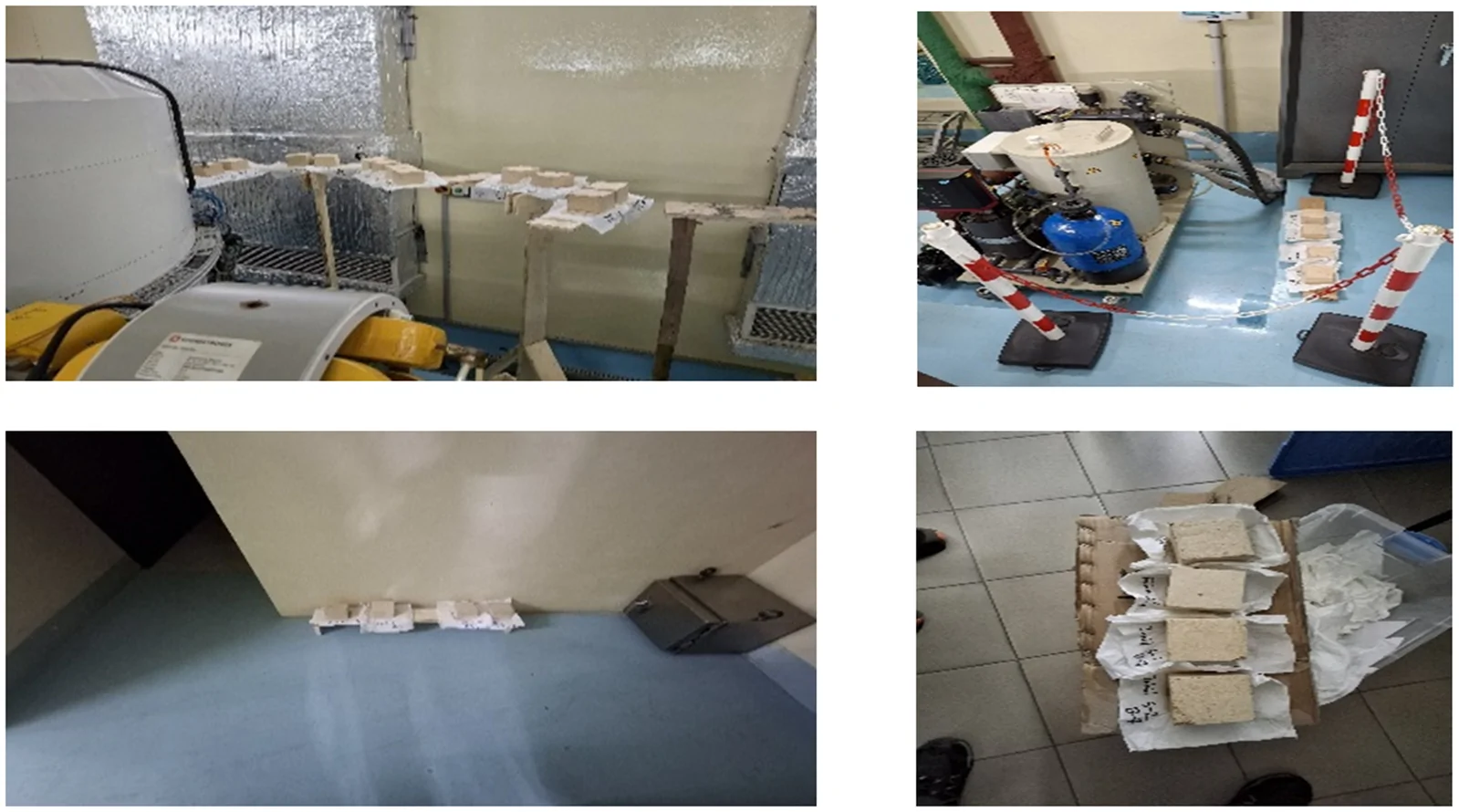
Sample kept in the vault room (a-c) and the physics department of CUET (d).

**Section-3(Sample analysis by** HPGe): The Radioactivity level in mushroom blocks were determined using gamma-ray spectrometry setup equipped with a vertical High Purity Germanium (HPGe) (Model No.Gx-4018) operated with Genie 2000 software. The detector has relative efficiency of 40%. A bias high voltage power supply is required to supply high voltage to the detector through the preamplifier. A power supply of 3000 V is adequate for the operation of germanium detector. This system included a lead- steel shield, preamplifier, a linear amplifier, high voltage power supply, a multichannel analyzer and a printer which is shown in Fig 5. The energy calibration of a germanium detector system is made by measuring point sources like Cs-137, CO-60 and Eu-152 with known activity and well-defined energies within the energy range of interest, usually 60 keV to 2000 keV. An accurate efficiency calibration of the system is necessary to quantify radionuclides present in a sample. Standard source Eu-152 is mixed with mushroom powder for counting efficiency calibration of High Purity Germanium (HPGe) and performs efficiency curve which is shown in Fig 6.

**Fig 5:**
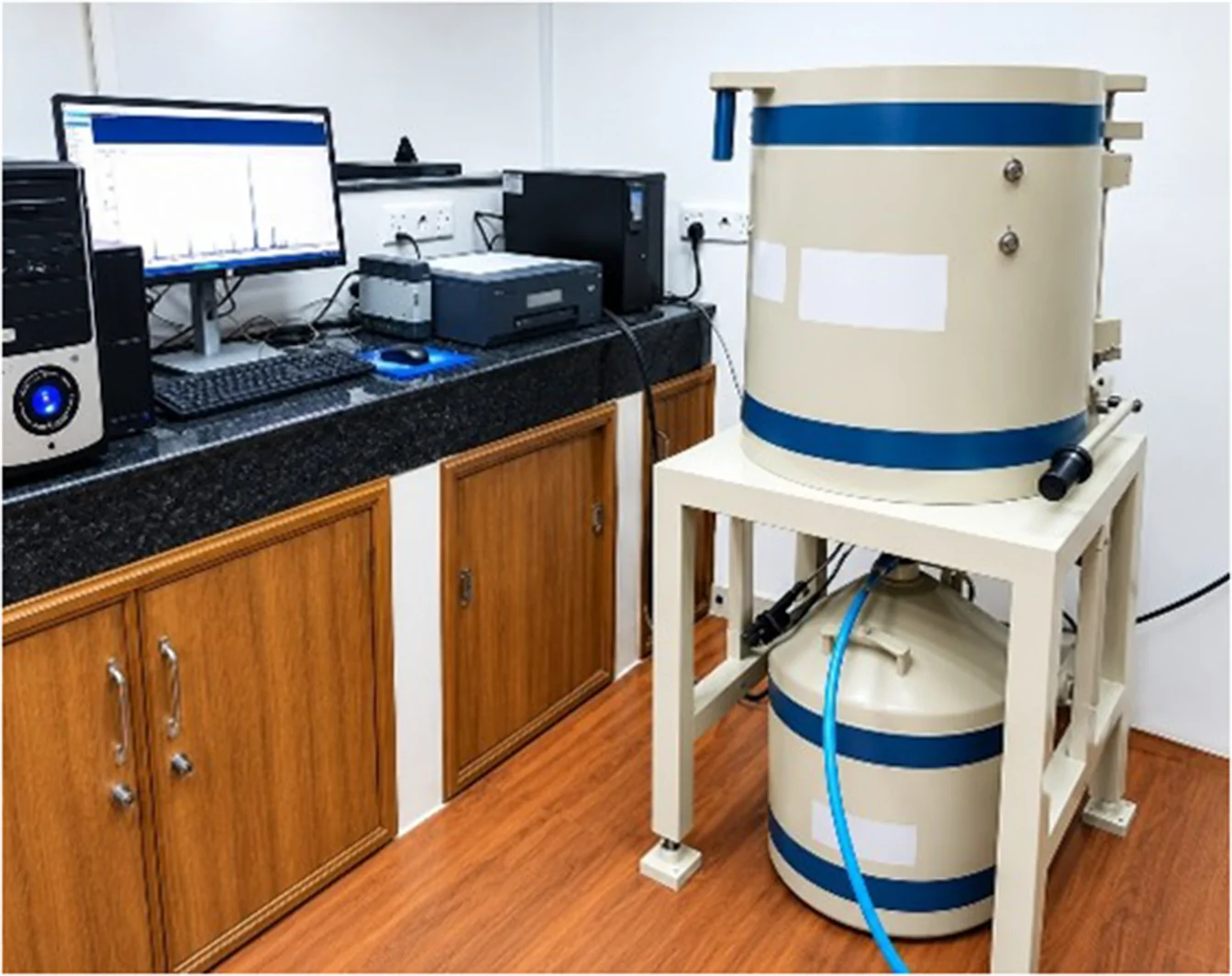
High purity germanium detector.

**Fig 6:**
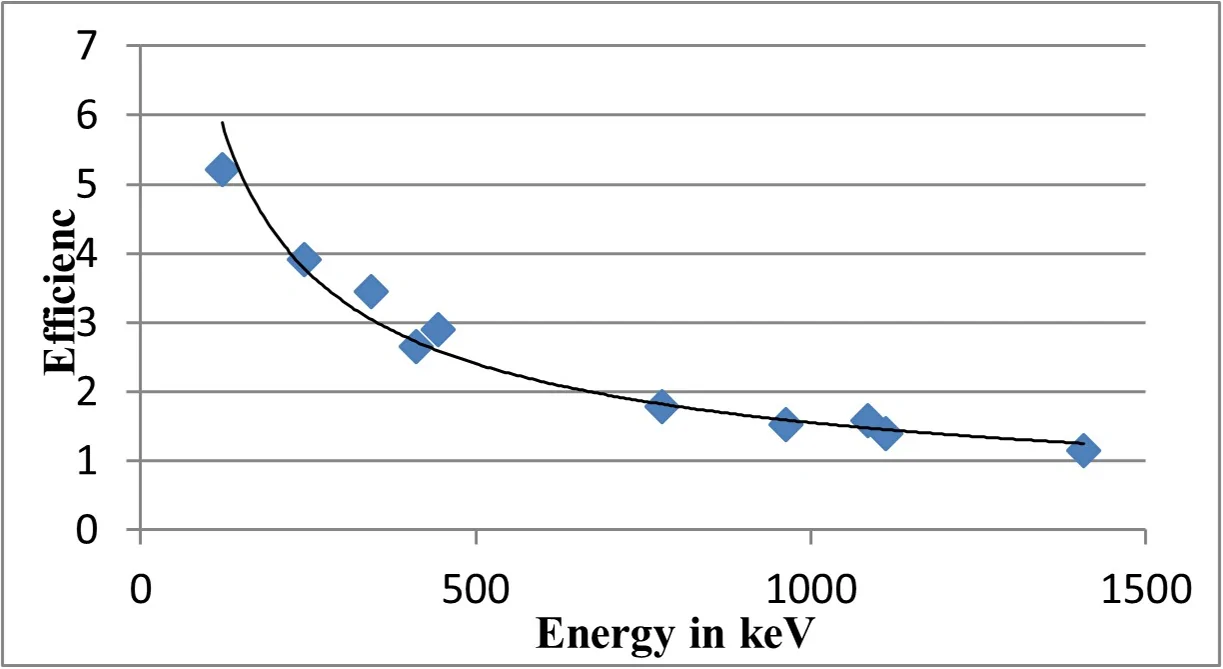
Energy vs efficiency curve. (Courtesy: Atomic Energy Centre, Dhaka).

## 3. Results & discussion

### 3.1. Structural Analysis by the XRD spectra

The structural analysis of the studied mushrooms has been performed by the XRD spectra (Rigaku Miniflex 600, CuKα) as shown in Fig 7. It is shown that crystallinity in the PC mushroom is higher than that of PO mushroom. The absorbance sharp peak would be expected in the PC at particular energy. However, the amorphous nature in the wide range is shown in the PO mushroom as expected in the natural mushroom’s samples (Fig 7).

**Fig 7:**
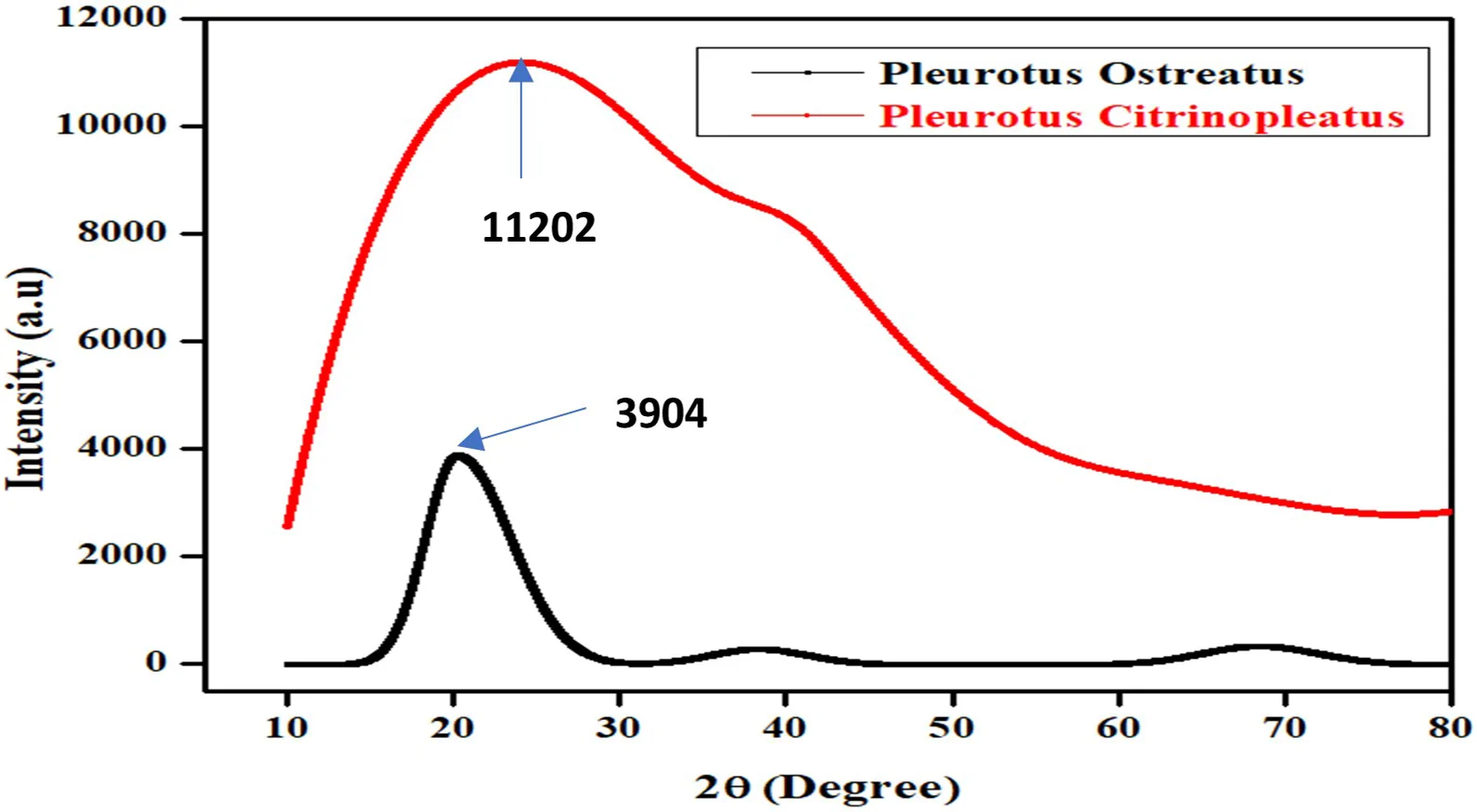
The XRD spectra of *Pleurotus Ostreatus* (PO) and *Pleurotus citrinopleatus* (PC) mushroom.

The Williamson-Hall (W-H) equation (1) is a simplified version of the Scherrer equation that connected the peak broadening (β) to the cryatallite size (D) and the internal lattice strain (ε). The formula represents


$$\begin{array}{c} \beta \text{cos}\theta =\frac{K\lambda }{D}+\text{4}\varepsilon \text{\textasciitilde{}}sin\theta \end{array}$$ (1)

where *k* is a dimensionless constant known as the shape factor (value assumed as 0.9), β is the peak’s full width at half maximum (FWHM), and θ is the Bragg angle. Simplified from equation 1 is D is equal to


$$\begin{array}{c} D=\frac{\text{0}.\text{9}\lambda }{\beta \text{cos}\theta } \end{array}$$ (2)

where λ is the radiation wavelength and β and θ are the Bragg angle and, e.g.,ral breadth, respectively. For the PO mushroom, peak at the value of 2θ = 20.9258 and the value of β is 6.02003. The crystallite size is estimated (using the **Scherrer equation** rather than a full Williamson–Hall analysis) and that is D = 1.34 nm.

### 3.2. Morphological study

The FESEM images of Pleurotus ostreatus (PO*) and* Pleurotus Citrinopleatus (PC*) are depicted in th*e Fig 8(a–f) *respectively at room temperature at* different magnifications. It is seen from the SEM images that all samples alike and agglomerated particle-like surface morphology are observed. The surface appears rough and folded at lower magnification (×5000), indicating a fibrous and layered cell wall structure made of chitin and polysaccharides. At medium magnification (×10000), the texture becomes more porous and sponge-like, showing interconnected voids that suggest air channels and nutrient pathways within the fungal tissue. At higher magnification (×30000), nanoscale fibrils and granular features are visible, representing the chitin microfibril network and protein–polysaccharide matrix that form the mushroom’s nanoscale architecture. The elemental analysis of the mushroom samples is performed by the EDS (energy dispersive spectroscopy). The elements are detected as carbon (C) and oxygen (O), confirming the organic, biopolymer nature of the fungal material. Minor elements such as Nitrogen (N), Sulfur (S), potassium (K), phosphorus (P), and calcium (Ca) are also be sensed as shown in Fig 9(a–d), indicating trace minerals or nutrient absorption. The percentages of the detected elements are summarized in the Table 1 and 2. Its structural and compositional features clearly make it a superior bio-adsorbent and radiation-shielding material, which is consistent with its possible application in nuclear and environmental studies [22,24].

**Table 1:** Various elements of *Pleurotus Ostreatus* (PO) mushroom.

Element	At. No.	Netto.	Mass [%]	Mass Norm. [%]	Atom [%]	Abs. error [%] (3 sigma)
**C**	6	12838	50.23	50.23	57.24	5.61
**N**	7	116	1.69	1.69	1.65	1.12
**O**	8	8356	48.04	48.04	41.09	6.07
**S**	16	18	0.04	0.04	0.02	0.17
		Sum	100.00	100.00	100.00	

**Table 2:** Different elements of *Pleurotus Citrinopleatus* (PC) mushroom.

Element	At. No.	Netto	Mass [%]	Mass Norm. [%]	Atom [%]	Abs. error [%] (3 sigma)
**N**	7	53	0.92	0.92	0.88	0.83
**C**	6	16193	61.25	61.25	67.93	6.75
**O**	8	5826	37.10	37.10	30.89	4.96
**S**	16	278	0.73	0.73	0.30	0.27
		Sum	100.00	100.00	100.00	

**Fig 8:**
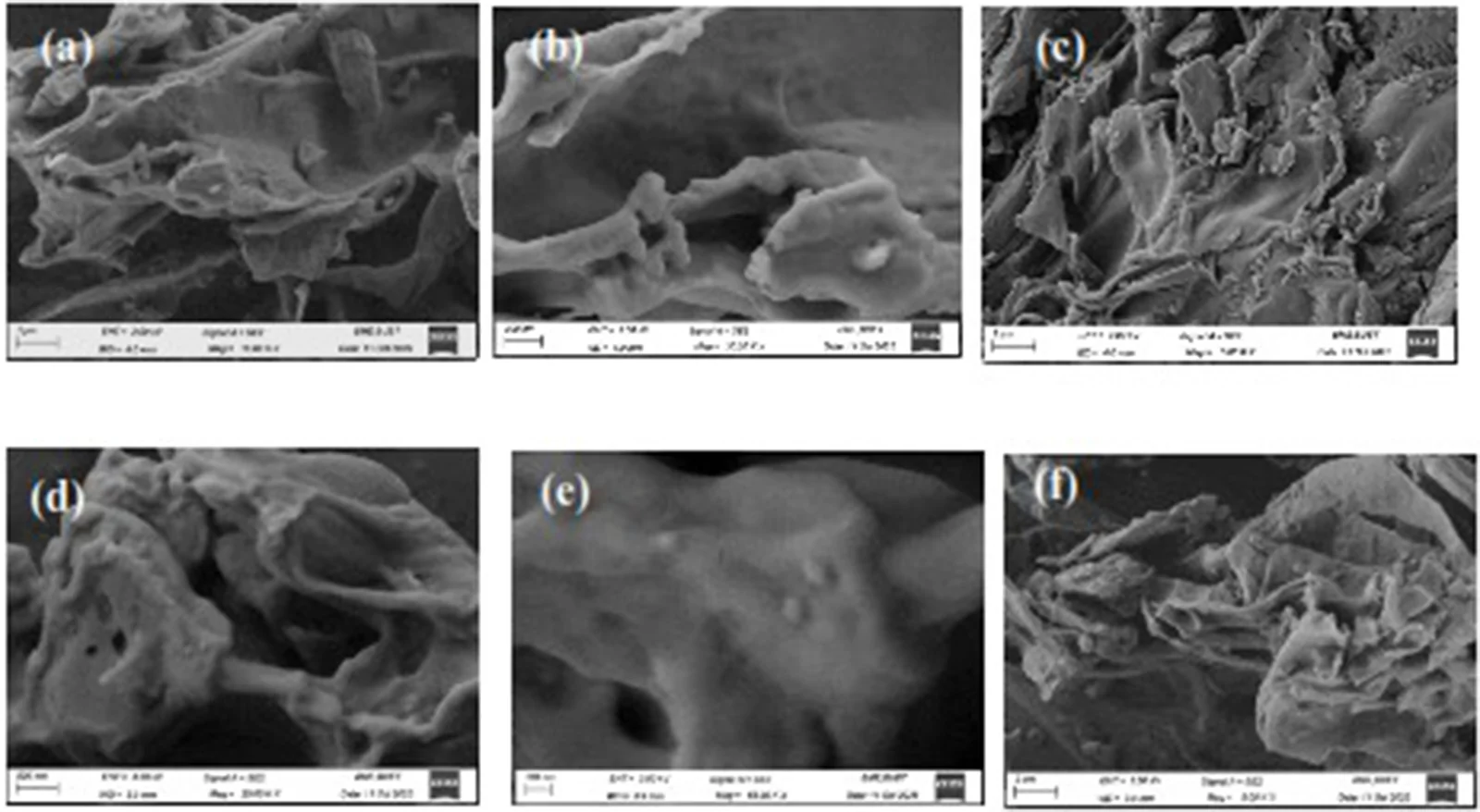
The FESEM images of *Pleurotus Ostreatus* (a-c) and *Pleurotus citrinopleatus* (d-f) mushroom.

**Fig 9:**
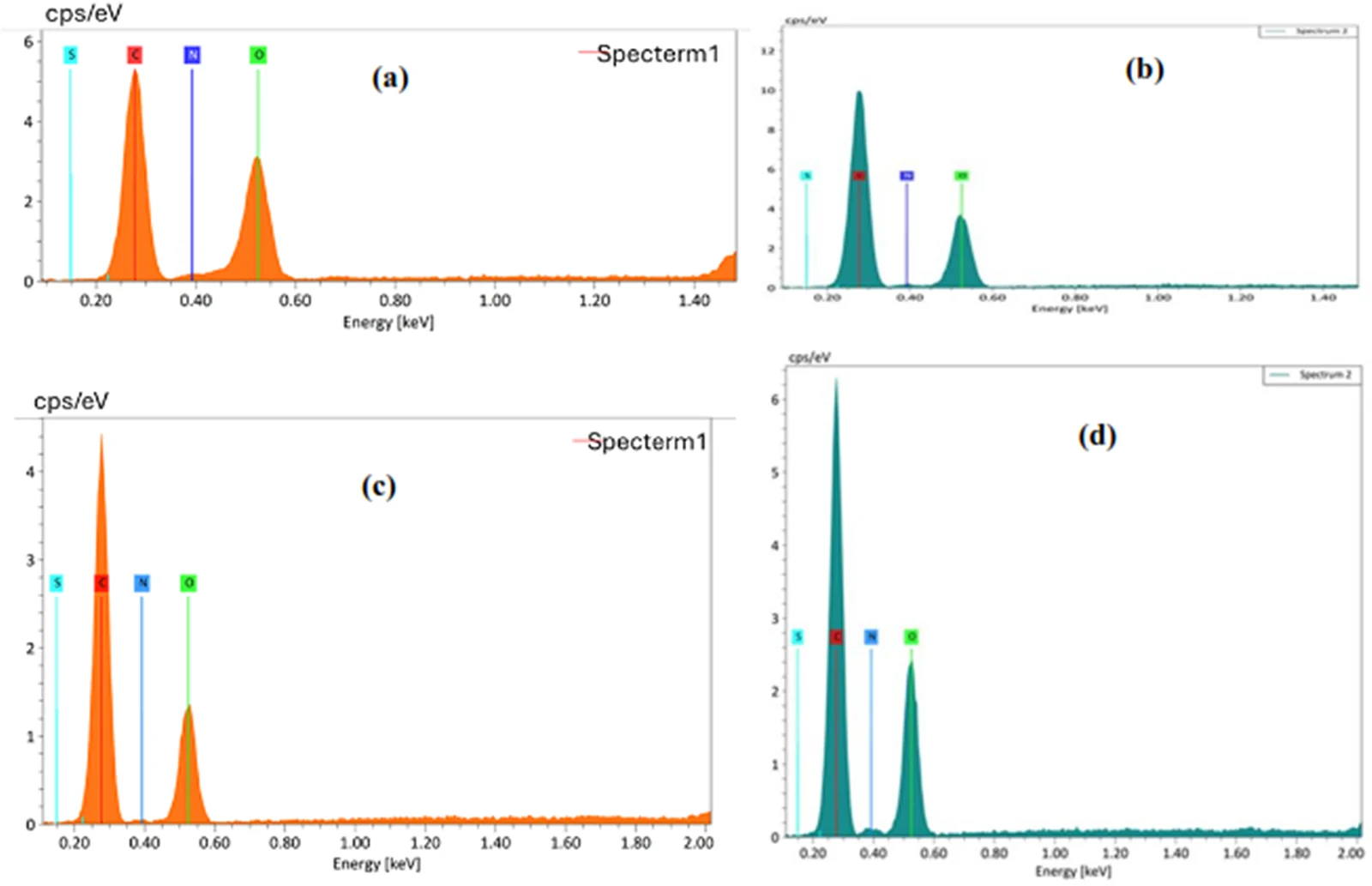
EDX spectra of *Pleurotus Ostreatus* (PO) and *Pleurotus citrinopleatus* (PC) mushroom.

The EDX spectra shows that the well-known elements S, N, C and O are dominant in the samples. The amount of these elements is summarized in the following table.

### 3.3. Functional group analysis

Fourier Transform Infrared (FTIR) analysis identifies and understands chemicals by examining their interaction with infrared light and measuring light absorption at various wavelengths by chemical bonds. The FTIR spectra of mushrooms at room temperature are depicted in Fig 10. The prominent peaks are identified between 450 cm^-1^ and 3850 cm^-1^. The prominent peak of S-O group is identified in the samples. The N-H and O-H groups are detected between 2800 cm^-1^ and 3000 cm^-1^. Moreover, the C = C = O, C-H and C = N are found to be at 2000, 1359 and 1619 cm^-1^, while S-O and C-O functional groups are found at 1019 and 1199 cm^-1^. It is noted that PC mushroom possesses one more functional group (M-O) at 504 cm^-1^.

**Fig 10:**
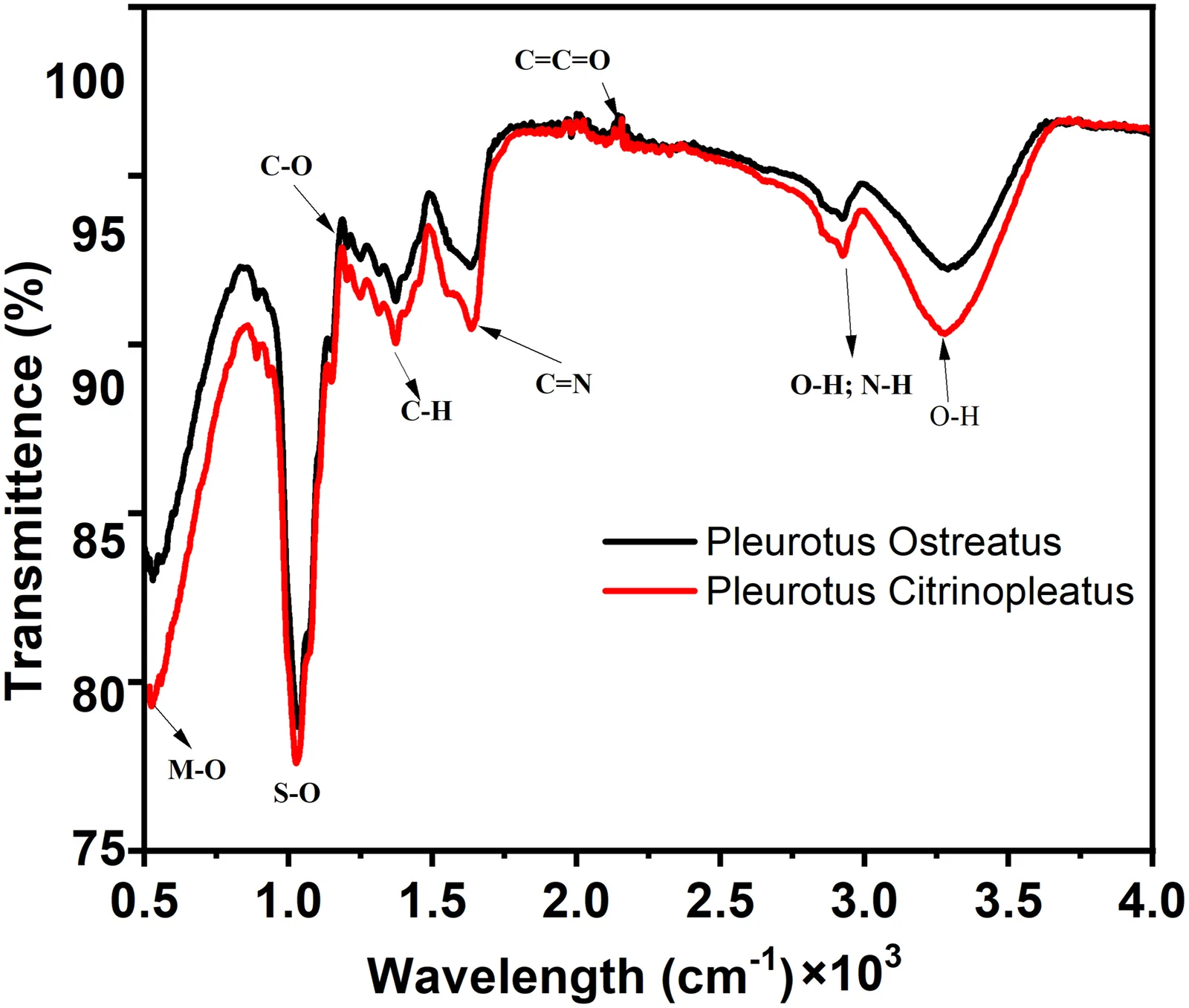
FTIR spectra of *Pleurotus Ostreatus* (PO) and *Pleurotus Citrinopleatus* (PC).

### 3.4. UV-vis spectra absorption analysis

The energy dependent absorbance spectra of *Pleurotus Ostreatus* (PO) and *Pleurotus Citrinopleatus* (PC) have been performed using UV-vis spectroscopy (LAMBDA 1050, PerkinElmer-USA) as shown in Fig 11. The absorption peaks are perceived for the PO at 244 and 274 nm indicate the presence of certain chemical elements or compounds that absorb at these wavelengths. These peaks most likely reflect the molecular orbitals of the chemicals found in the mushroom, especially those associated with the electronic transitions of different components (such conjugated systems or aromatic structures) that consistent with the observation by the EDX. However, in the case of PC sample exhibits absorption characteristics at 284 nm, 320 nm, and 371 nm, which point to particular interactions within the sample. Carbon (C) edges that are typical of substances with organic carbon structures, are frequently blamed for the peak at 284 nm. The peak at 371 nm represents the pre-edge or near-edge region between the C and N (nitrogen) edges, whereas the feature at 320 nm may represent post *k*-edge carbon atoms (where there is a higher energy transfer of electrons).

**Fig 11:**
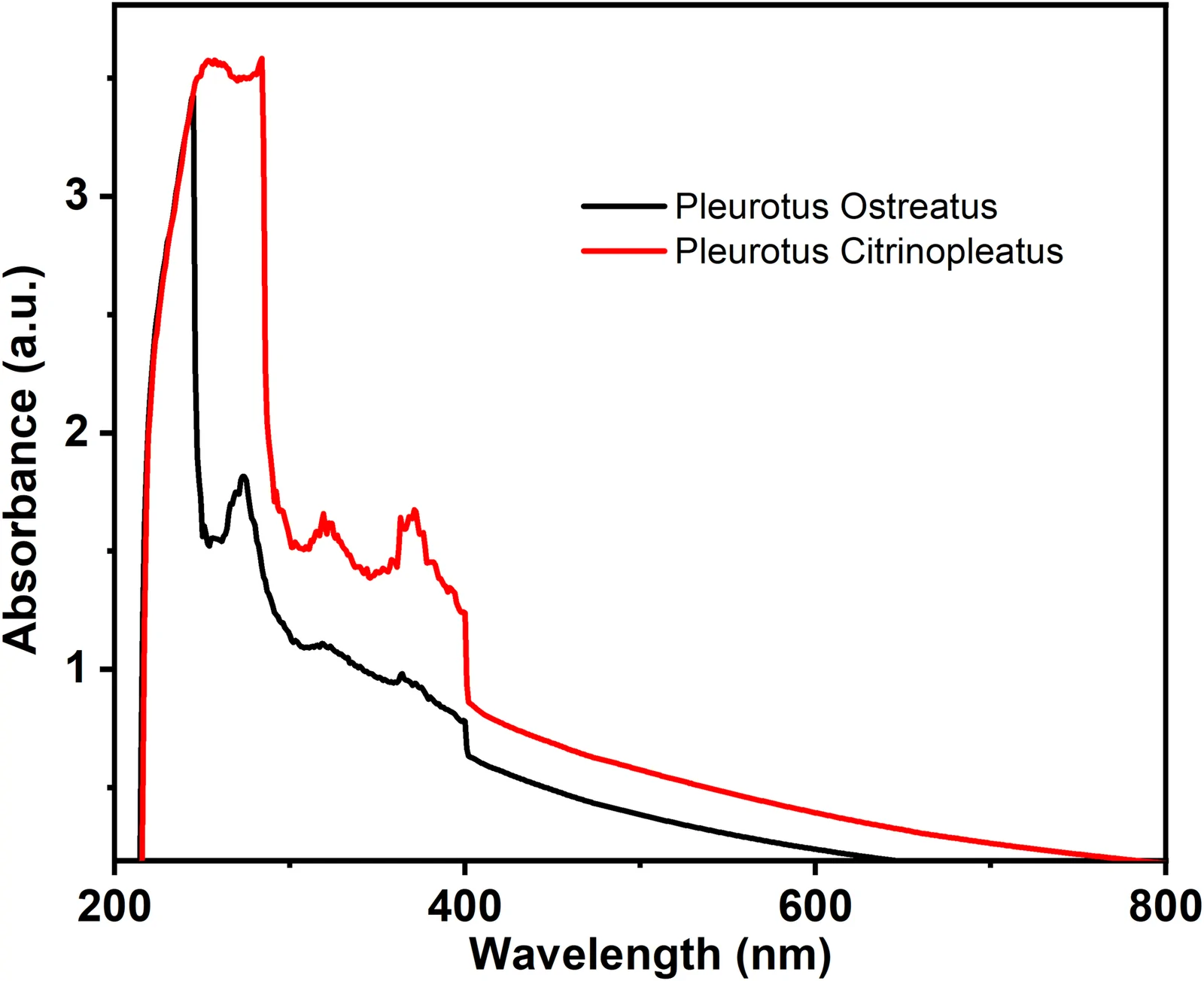
Absorbance spectra obtained from the UV-vis diffuse reflectance.

The materials derived from mushrooms were analyzed for electronic absorption using UV-Vis diffuse-reflectance spectroscopy. The absorption characteristics are attributed to electronic transitions in organic molecules, providing insights into their electronic structure. However, these findings do not directly relate to neutron moderation or capture capabilities, as the UV-Vis data do not quantitatively measure neutron-shielding performance, serving only as physicochemical characterization.

### 3.5. Neutron absorbance spectra analysis by the HPGe detector

Activation products detected after neutron exposure show that mushrooms partially absorb and moderate neutrons, contrary to expected minimal activation if they were ineffective shields. The hydrogen-rich organic matrix, along with nitrogen and sulfur, inhibits neutron flux. Measurable radioactive traces following cyclotron irradiation are used by HPGe detector measurements to demonstrate neutron–material interaction [32–34]. These findings show the mushroom blocks’ potential for neutron moderation and capture; however, specific neutron transmission experiments are needed to determine direct attenuation coefficients. A Typical spectrum of Mashroom Block-07, Sample-02 using HPGe detector is shown in Fig 12.

**Fig 12:**
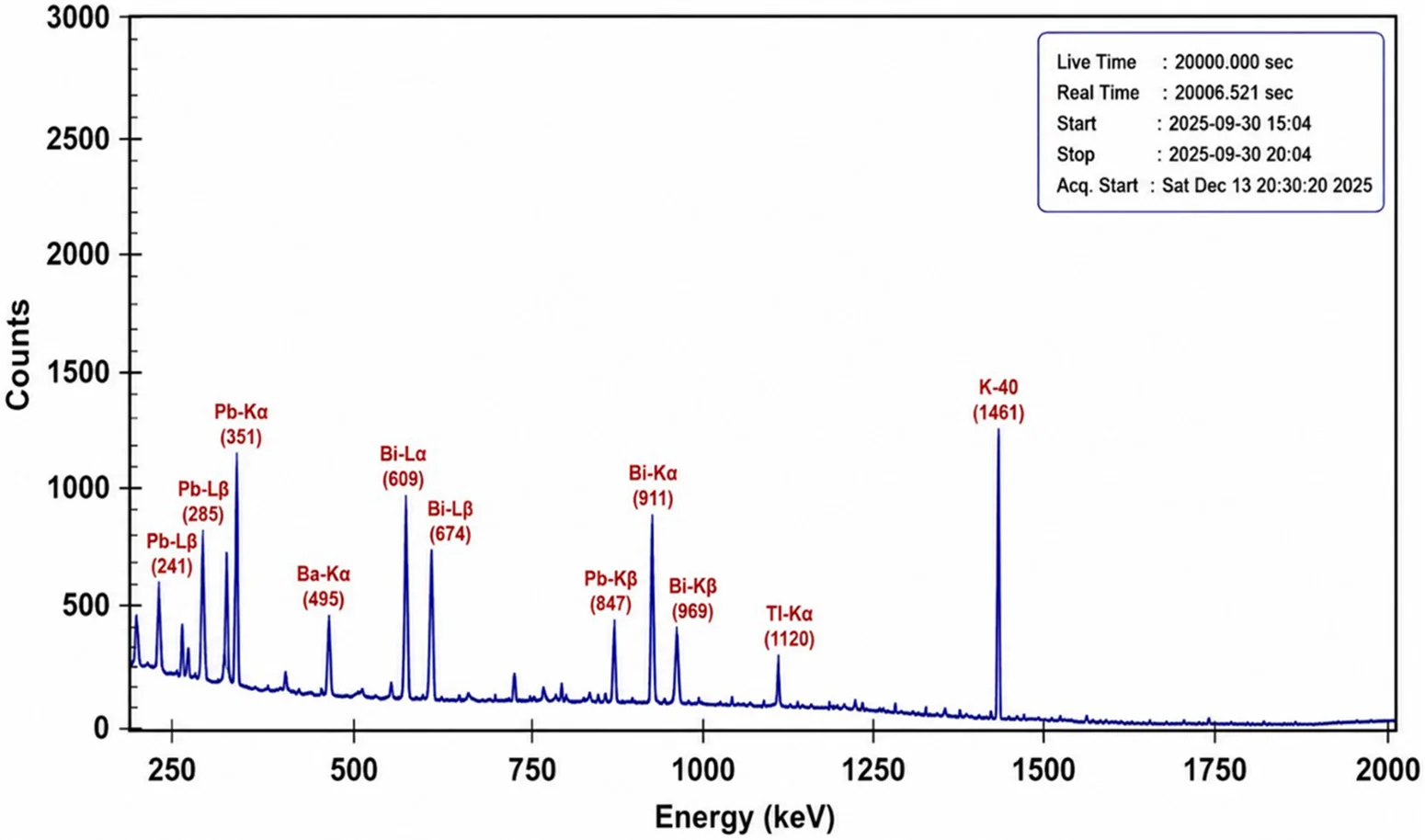
Typical spectrum of Mashroom Block-07, Sample-02 using HPGe detector.

After 70 days of exposure, the mushroom samples are measured by the HPGe detector to estimate the neutron absorbance efficiency of that samples in the cyclotron vault room and compared these results with radiation absorbs by the samples were placed outside the vault room. It is observed that the PO mushroom blocks show measurable natural radioactivity, with ^40^K clearly dominating the activity concentrations in every sample (~28–38 Bq/kg). The highest K-40 level occurred in the sample block 5, while Blocks 4 and 6 demonstrate comparatively lower values. Radionuclides in the U-238 and Th-232 decay series are presented at much lower levels, usually ranging from 3–8 Bq/kg. Blocks 2 and 7 consistently exhibit higher activities for both decay series, whereas blocks 3 and 5 showed the lowest concentrations. The findings indicate that K-40 is the primary contributor to total radioactivity in the mushroom-derived blocks, while uranium- and thorium-series radionuclides contribute only minor amounts. Despite identical exposure conditions, noticeable inter-block variability suggests non-uniform radionuclide accumulation or interaction within the blocks.

Almost similar trend observes after 70 days of cyclotron exposure, the PC mushroom blocks show measurable natural radioactivity. The **dominant radionuclide** element ^40^K **determines** in every block (∼25–38 Bq/kg), where block-5 represents the highest activity. **Uranium- and thorium-series radionuclides are also presented with much lower levels**, typically around 3–6 Bq/kg for the ^238^U series and 4–7 Bq/kg for the ^232^Th series. Although all blocks experienced identical exposure conditions, **clear inter-block variability** was observed, indicating non-uniform radionuclide accumulation within the mushroom-derived blocks.

The HPGe detector results depict that the amount of K-40 is higher in block number 5. When neutrons are released from the Cyclotron machine, even though all the neutrons hit directly, some neutrons are scattered in the vault room. These neutrons are later accumulated immediately the vault room door passage and increases the neutron density at that place. The block-5 sample places at that position results higher absorbance of neutron accumulation are observed by measuring K-40 as shown in Figs 13 and 14.

**Fig 13:**
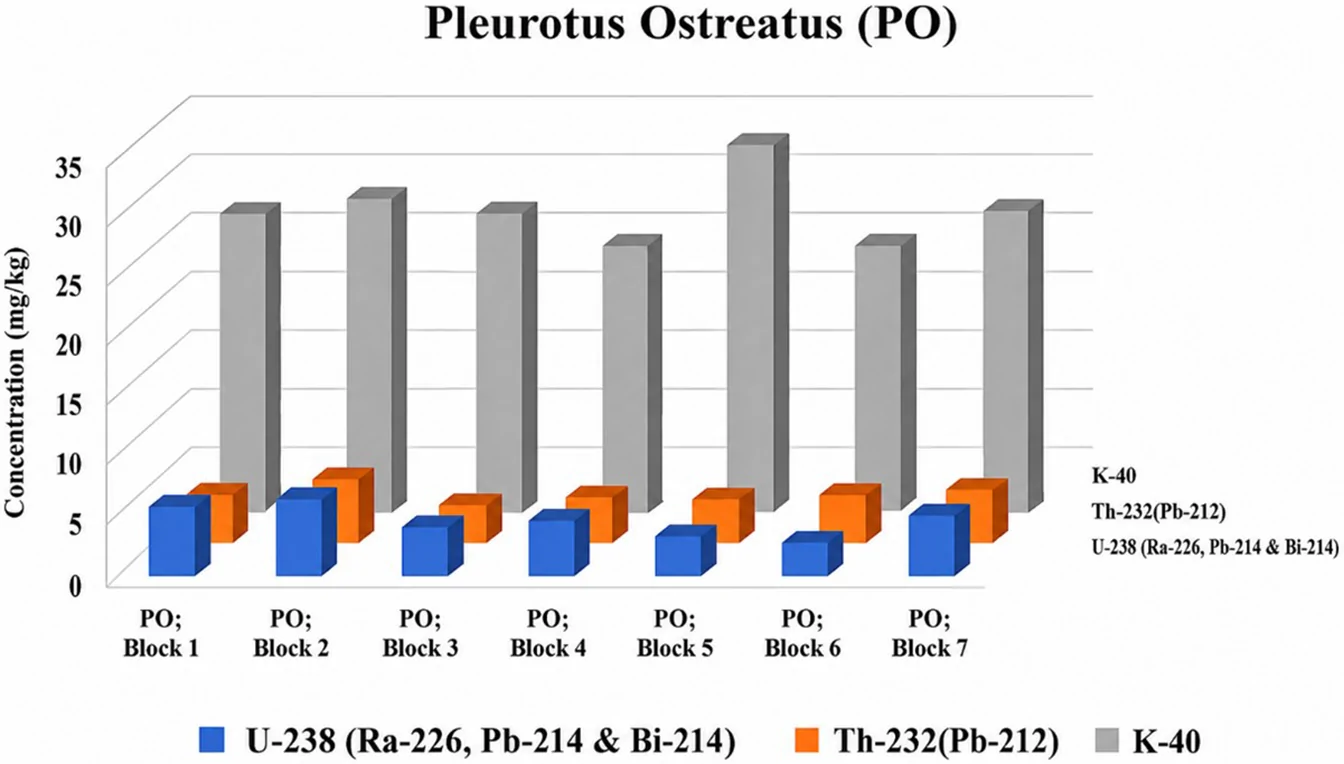
The bar diagram of radioactivity concentration of U-238,Th-232 and K-40 in *Pleurotus Ostreatus* (PO) Mushroom.

**Fig 14:**
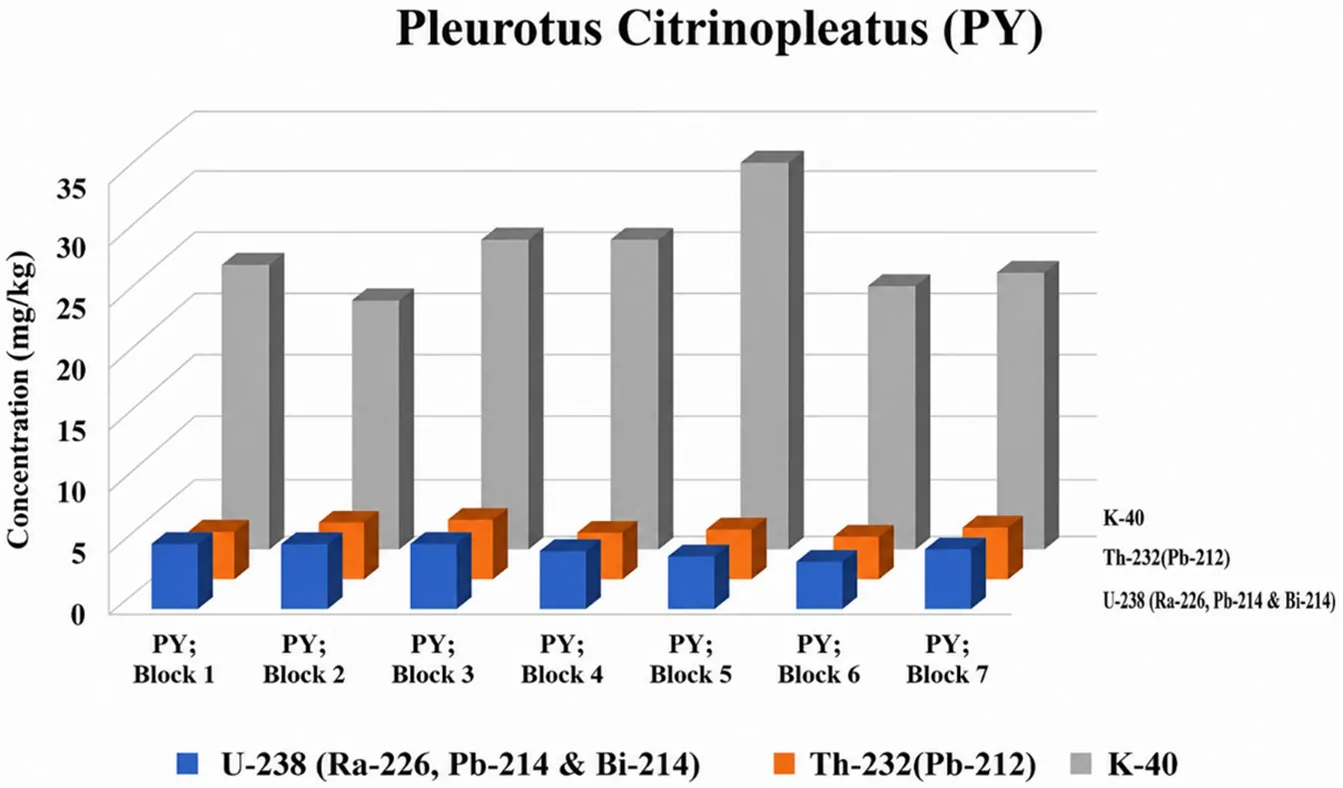
The bar diagram of radioactivity concentration of U-238,Th-232 and K-40 in Pleurotus Citrinipleatus (PC) Mushroom.

The proposed neutron interaction mechanisms in shielding materials produced from mushrooms are shown schematically in Fig 15. The process starts with an initial input stage, which is followed by a pre-processing step. The system moves on to a core processing module, where the mechanism splits into two different directions. Every path denotes a unique processing approach that results in the corresponding output.

**Fig 15:**
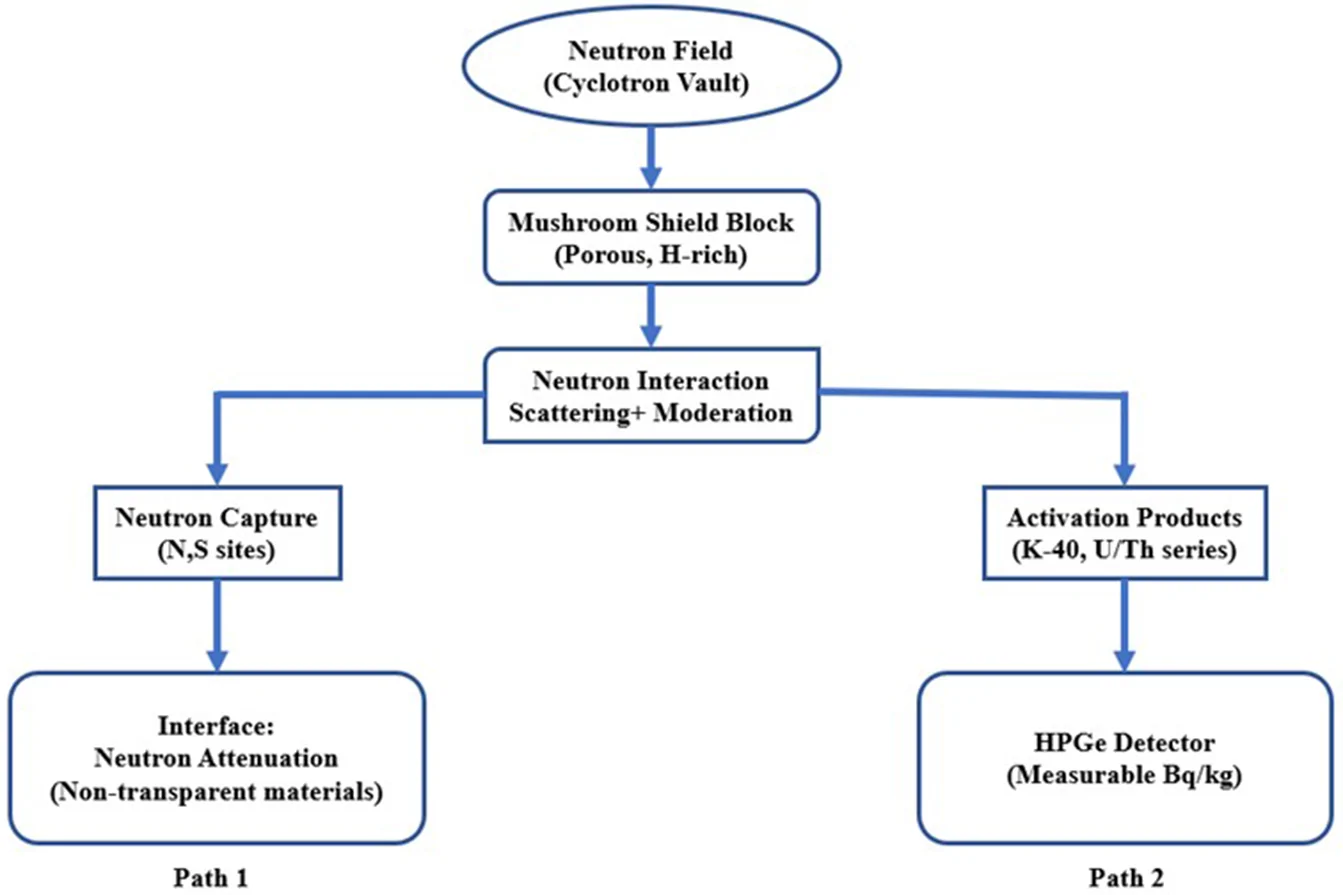
The proposed mechanism of neutron absorbance by the mushrooms and that are measured by the K-40 using HPGe detector.

Both vault-exposed and control mushroom blocks showed detectable gamma activity, mainly from naturally occurring 40K, with some contribution from 238U and 232Th decay series. Variations in 40K activity cannot be fully explained by thermal-neutron capture, due to insufficient paired HPGe measurements and lack of independent quantification of neutron-specific activation products. Therefore, the results should be seen as evidence of geographical variation in gamma activity from samples in different radiation environments, rather than definitive isotope-specific evidence of neutron activation. Future research will incorporate pre-irradiation baseline spectra, matched non-irradiated controls, timely post-irradiation HPGe measurements, and decay-time analysis of activation products such as 42K. Additionally, complementary beta counting will be necessary for products like 32P.

**Comparison with conventional neutron-shielding materials:** The study focused on proving the concept of neutron interaction with compressed mushroom-derived materials, rather than functioning as a typical transmission experiment. Current HPGe data indicate that the compressed PO and PC mushroom blocks interact with the cyclotron neutron field, complicating the calculation of the transmission factor, effective neutron removal cross-section. In literature, fast-neutron removal cross-section values are approximately 0.09 cm^-1 for concrete and 0.11 cm^-1 for paraffin wax and polyethylene, though these values may vary based on neutron spectrum, density, composition, and experimental setup. Concrete with 20 weight percent B4C exhibits an attenuation coefficient of 0.299 cm^-1 under specific Am–Be irradiation conditions, highlighting its enhanced thermal-neutron capture capabilities. The findings suggest that the data should be interpreted as evidence of neutron-interaction potential, rather than direct comparisons with established shielding materials. A precise evaluation will necessitate direct measurements under consistent irradiation conditions.

## 4. Conclusions

The shielding effectiveness by absorbing neutrons of *Pleurotus Ostreatus* (PO) and *Pleurotus Citrinopleatus* (PC) mushrooms in the cyclotron vault room has been performed. The structural and morphological analysises are studied using XRD, FESEM, FTIR, and UV-vis spectroscopy. The XRD pattern of PO reveals a single broad diffraction peak, suggesting ultra-fine nanocrystalline domains with an average crystallite size of 1.34 nm, while PC shows no distinct peaks, indicating its amorphous nature and a lack of long-range atomic order, leading to increased neutron scattering randomness and interaction probability within the material. The FESEM micrographs of PO and PC demonstrate a highly irregular, porous, and fibrous surface morphology, which is beneficial for neutron shielding applications by increasing neutron scattering paths and enhancing interaction probabilities within the material. EDX value shows for *PO* and *PC* mushrooms, that the species of mushrooms contain carbon (50–61)%, which acts as a hydrogen-rich organic matrix and provides neutron moderation. Oxygen (37–48)%, which acts as elastic scattering, and nitrogen (0.9–1.7)%, which acts as a thermal neutron absorber and sulfur (0.04–0.73)%, which acts as a neutron capture contributor. The electronic absorption features of mushroom-derived materials, analyzed via UV-Vis spectroscopy, should not be viewed as direct indicators of neutron capture or moderation. Neutron interactions, influenced by energy and elemental or isotopic composition, govern these processes. Additionally, while contributions to neutron capture are determined by isotope-specific nuclear cross-sections, the hydrogen-rich organic matrix may contribute to neutron moderation through elastic scattering. The absorption characteristics align with findings from FTIR and EDX, indicating the presence of complex organic carbon systems. The presence of O–H and N–H groups enhances hydrogen density, while C–N and S–O bonds contribute to thermal neutron capture sites. The evaluation of neutron interaction in compressed mushroom blocks showed measurable natural radioactivity, mainly K-40, ranging from 25 to 38 Bq/kg, detected via HPGe gamma spectroscopy after 70 days in a medical cyclotron facility.

The ultimate bulk density and residual moisture content of the compressed PO and PC blocks were not quantitatively measured prior to and following the 70-day cyclotron-vault exposure, which is a drawback of the current study. Furthermore, mechanical characteristics and post-exposure dimensional stability were not quantitatively assessed. As a result, even though the current HPGe tests show neutron contact with the compressed mushroom blocks, it is currently impossible to quantify the impact of density and moisture fluctuation on their long-term neutron-moderation ability. Future research will detect equilibrium moisture content, calculate bulk density from observed mass and volume, and evaluate mass loss, dimensional change, compressive strength, and microstructural properties both before and after irradiation. These observations will provide a direct correlation between hydrogen density and structural stability and neutron attenuation performance.
